# Comparative Clinical Study of Two Tooth Bleaching Protocols with 6% Hydrogen Peroxide

**DOI:** 10.1155/2009/928306

**Published:** 2010-02-04

**Authors:** Jesús Oteo Calatayud, Paloma Mateos de la Varga, Carlos Oteo Calatayud, María José Calvo Box

**Affiliations:** ^1^Department of Conservative Dentistry, School of Dentistry, University Complutense of Madrid, 28040 Madrid, Spain; ^2^Department of Conservative Dentistry, School of Dentistry, University Rey Juan Carlos, 28922 Alcorcón, Madrid, Spain

## Abstract

*Objective.* The objective was to compare the clinical efficacy of two different tooth bleaching protocols after 1 and 2 weeks of treatment with an over-the-counter paint-on gel containing 6% hydrogen peroxide. *Material and methods.* Sixteen volunteer patients (minimum shade A2 or darker on maxillary teeth) were selected to participate in this randomized, single-blind (examiner-blinded), single-center, 2-group clinical trial using a divided mouth model. The product was applied in our clinic to one hemi-arch (Group I) in each patient at two sessions one week apart, making five applications at each session (separated by 10 min intervals). The patients themselves applied the product once a day for 10 days in the other hemiarch (Group II). Efficacy was measured according to the Vita Classical shade guide at baseline and at one and two weeks. Differences between groups (office-treated vs. home-treated hemiarches) were tested by repeated-measures analysis of variance. *Results.* Significant (*P* < .05) differences in shade values were detected between pre- and post-bleaching in both groups. The two groups did not significantly differ in tooth shade at the end of the treatment. *Conclusions.* Treatment with 6% hydrogen peroxide gel using the paint-on system shows significant clinical efficacy whether applied by clinicians or by the patients themselves.

## 1. Introduction

There has been a major increase in demand for dental esthetic treatments, including tooth whitening. One recent change in vital tooth bleaching has been the development of home treatments, which are available in some countries without a prescription [1]. A growing number of products have entered the market, including low-cost over-the-counter bleaching systems of different concentrations that are easy to apply. They do not require a customized tray, therefore patients no longer feel the need to be treated by a professional [2].

Peroxide is the most widely used bleaching agent in these treatments, in the form of carbamide peroxide or hydrogen peroxide [3]. Several over-the-counter bleaching systems are available with different application methods. One uses generic trays to apply a whitening gel but must be administered with care because of the possibility of soft tissue lesions, malocclusion, and a poor outcome [4]. In another approach, polyethylene strips on which hydrogen peroxide is uniformly distributed are directly applied on the vestibular surface of teeth. Trays are not required and they are easy and comfortable to use [5], but they adapt poorly to malpositioned teeth [6]. In some more recent varnish systems, the gel is painted on the outer surface of the teeth and the peroxide is then slowly removed until finally eliminated by tooth brushing [7–9]. One advantage of this technique is that it can be applied at any time of day, although the possibility of prematurely removing the peroxide by lip or tongue movements represents a major drawback. 

The clinical efficacy of systems is expected to be greater with longer contact time between the agent and tooth surface. Accordingly, products applied with the paint-on system would be more effective if they were less susceptible to early removal by lip, cheek, or tongue movements. 

The main objective of the present study was to assess, in a clinical trial, the whitening efficacy of a bleaching varnish of 6% hydrogen peroxide applied for 2 weeks on the upper-front teeth, conducting a comparative clinical study of two application protocols: in-office treatment by the clinician with totally isolated teeth and no contact with soft tissues versus home treatment by the patient with no soft tissue isolation.

## 2. Material and Methods

An initial sample of 20 patients aged from 18 to 30 years was selected from patients who came to our School of Dentistry for tooth bleaching. A randomized, single-blind (examiner-blinded) trial was conducted using a divided mouth model with two study groups. The examiner and all staff were blind to the treatment assigned to each hemiarch throughout the study. The study protocol was approved by the Clinical Research Ethical Committee (Code: P-09/073) of Madrid Clinical Hospital (Spain). After the treatment was explained to the patients, they signed a detailed informed consent form that outlined all procedures and defined the alternatives. All participants were offered a supplemental bleaching treatment after the end of the study. 

Study inclusion criteria (evaluated at a first examination) were as follows: age ≥18 years, absence of gingival recession or restorations in upper-front teeth, good oral hygiene and gingival health, no previous tooth bleaching procedure, tooth shade of A2 or above on the Vita Classical Shade Guide scale (Vident, Brea, Calif, USA.) ordered by value, and, in the case of females, not being pregnant or in breast-feeding period.

Four patients from the initial sample did not come back for the first study session; therefore, the final study sample comprised 16 patients.

For the given effect size (population mean difference of 2.0 SD of change = 2.3) and sample size (16 pairs), the alpha (0.05 2-tailed) power was 0.901 (SamplePower version 2.0). 

At study enrolment, all patients were given a Vitis medium toothbrush (Dentaid, Barcelona, Spain) and Fluor-aid 250 toothpaste (Dentaid) for use from two weeks before the treatment to the end of the study period. They were instructed to avoid any type of food and drink such as red wine, coke, curry sauce or mustard with colorants in their diet during the study period. 

**Figure 1:**
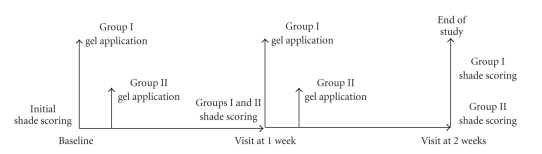
Treatment sequence.

Figure 1 summarizes the treatment sequence for the two study protocols. A divided mouth model was used, treating both hemiarches with 6% hydrogen peroxide VivaStyle Paint On Plus (Ivoclar Vivadent AG, FL-9494 Schaan/Liechtenstein). Each hemiarch was assigned, using a randomly generated numbers table, to one of two groups: Group I, for in-office treatment by a professional, and Group II, for home treatment by the patients themselves.

**Figure 2:**
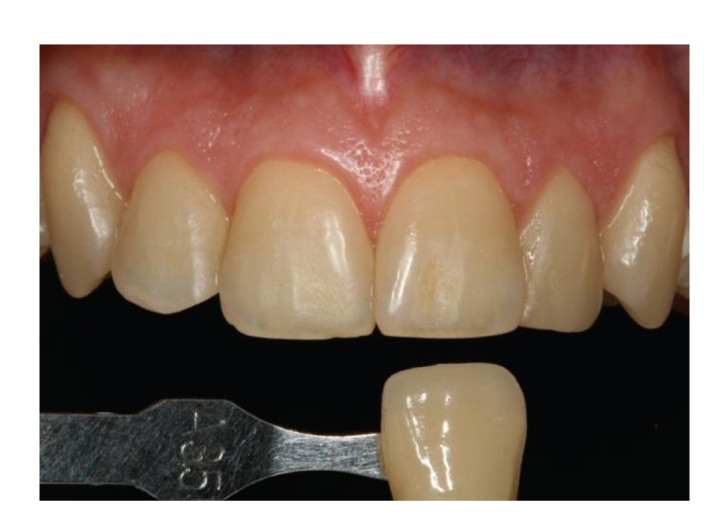
Shade scoring at baseline.

Before the bleaching treatments, baseline numerical shade values were obtained for central incisors and canines of both hemiarches in accordance with the Vita Classical Shade Guide (Figure 2). Shade tabs were arranged in the sequence recommended by the manufacturer, assigning each tab with a number from 1 to 16 (B1, A1, B2, D2, A2, C1, C2, D4, A3, D3, B3, A3.5, B4, C3, A4, C4). The shade was recorded by two independent examiners not otherwise involved in the study (K coefficient = 0.85, standard error = 0.09), who were blinded to the materials used. They used the Demetron Shade Light system (KerrHawe S.A. Bioggio, Switzerland) to ensure the same light conditions (5500 K) for the shade scoring of all teeth. 

In Group I, soft tissues of the mouth, lips, and cheeks were protected before application of the hydrogen peroxide by using the Optragate system (Ivoclar Vivadent, Schaan, Liechtenstein). The teeth were then air-dried with the dental unit syringe before using the brush from the kit to apply 6% hydrogen peroxide varnish on the surface of teeth in the corresponding hemiarch, where it was maintained for 10 minutes before being removed with pressurized water. Another layer of varnish was then painted on and left for a further 10 minutes. This process was repeated five times in the same session. Immediately after this treatment session, the shade of the treated hemiarch was recorded, and the patient received instructions on the treatment to be conducted at home. In Group II, once a day for 5 consecutive days, patients had to lift their lips away from the teeth of the contralateral (untreated) hemiarch, dry the teeth with absorbent cellulose paper, apply the hydrogen peroxide varnish with the brush, and then wait for 30 seconds before closing their mouth. Patients were instructed not to eat or drink anything during the following 10 minutes.

**Figure 3:**
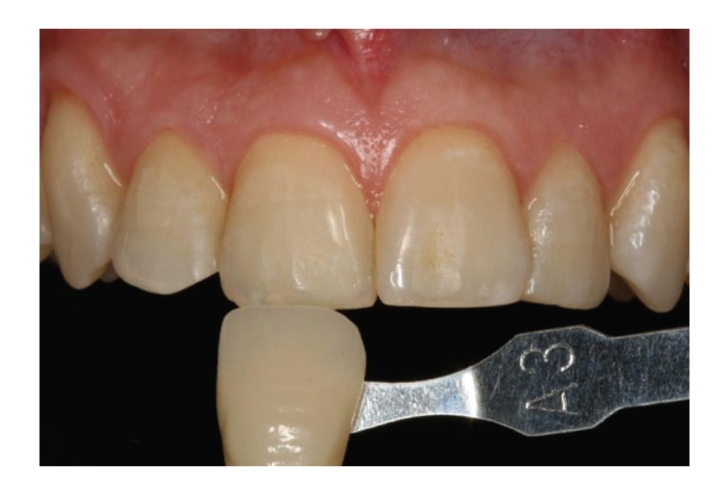
Shade scoring after 5 applications of treatment in both hemiarches.

**Figure 4:**
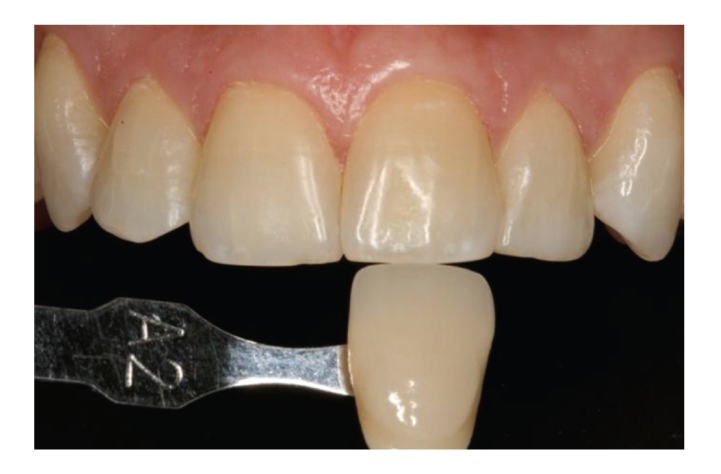
Shade scoring at end of study.

After this 5-day home treatment, the patients visited the dental clinic for a second time. The shade of the central incisors and canines in each hemiarch was recorded (Figure 3), and the clinical application protocol was repeated (5 consecutive 10-minute applications), followed by the home treatment protocol (10-minute application on 5 consecutive days). At the end of the study, after a total of 10 applications per hemiarch, the shade of the teeth under study was again recorded at the clinic by the same examiners (Figure 4).

The soft tissues of all patients were examined at the end of each clinical session, and they were asked about any possible adverse effects during the treatment, recording their responses on the corresponding data sheet.

### 2.1. Statistical Analysis

The Shapiro-Wilk normality test was first applied to evaluate the normal distribution of means. Paired Student's *t*-test was used to compare mean baseline values between treatment groups. A repeated-measures analysis of variance (ANOVA) was used to study interactions between treatment and time and to analyze the interaction of these two factors with tooth shade. A multiple comparisons analysis was performed *a posteriori *to study differences in mean values between baseline and at one and two weeks of treatment, adjusting the *P* value to the number of comparisons. In all statistical tests, *α* ≤ 0.05 was considered significant. SAS version 9.1.3 software (SAS Institute Inc., Cary, North Carolina, USA) was used for all data analyses.

## 3. Results

Sixteen patients (6 males and 10 females) completed the study protocol, with a mean age of 23.8 ± 2.8 years (range, 18–30 years). No adverse effects on soft tissue were reported by the patients or observed by the examiners.

Table 1 shows the tooth shade values (mean ± standard deviation) for each group at the three measurement time points. At baseline, the mean VITA score of Group I teeth was 7.5, and the mean score of Group II teeth was 7.3, a nonsignificant difference (*P* = .5278).

At one week, after the first 5 applications (in-office or home treatment), scores were significantly lower versus baseline in Group I (1.5 shades less, *P* = .0172) and Group II (1.7 shades less, *P* = .0053). 

After two weeks of treatment (10 applications), Group I showed a significant lightening versus baseline of 2.8 shades (*P* = .0001) and Group II a significant lightening of 2.5 shades (*P* = .0007). The shade scores of Group I (4.7) and Group II (4.8) did not significantly differ (*P* = .5636) at the end of study.

## 4. Discussion

Sample size was established for a minimum difference of two shades, because a smaller difference is virtually imperceptible. The total number of teeth was 32, analyzing 2 teeth (central incisor and canine) per patient. Central incisors and canines were selected in order to obtain a more homogeneous sample, because central and lateral incisors usually have the same shade and could, if considered together, distort the mean hemiarch scores [10, 11].

Visual assessment with shade guides, computer analysis of digital images, colorimetry, and spectrophotometry can all be successfully used to measure the color change of teeth in longitudinal tooth bleaching studies [12]. Digital images offer an objective shade difference value, but the light and positioning of the subject must be standardized and a robust mathematical transformation algorithm must be used [13]. We used the Vitapan Classical shade guide ordered by value, considered a valid and reliable method for color assessment [14], and applied in previous investigations [2, 11, 15, 16]. Other more sophisticated measurement systems are available [10, 17, 18] but do not offer a more reliable accuracy, since different color measurements can be obtained for the same tooth according to the positioning of the probe tip of the device [19]. Visual assessment is a subjective method in which tooth and shade guide are simultaneously observed under the same light conditions. The light system used in the present study ensured that the light projected on the arch was identical for all measurements.

**Figure 5:**
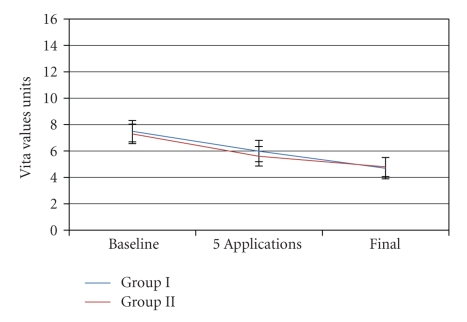
Treatment time course with each protocol.

A lightening of 2.8 shades was achieved with the in-office professional treatment and a similar lightening of 2.5 shades with the home treatment. Although both treatment protocols were effective, superior outcomes have been reported with the use of other systems (see below). A value of 1, corresponding to B1 (maximum luminosity), was obtained in only one case. However, outcomes may improve with longer treatment times [20], as indicated by the treatment time-course graph (Figure 5). The effectiveness may have been limited by the absence of a mechanical barrier to keep the product in the mouth for a longer time. Comparative studies have reported better outcomes for systems that maintain the product in the mouth (e.g., with strips) than for paint-on systems [21–23]. In the present study, the protocol applied showed very little influence on the efficacy, with no significant difference in outcomes at two weeks between an in-office professional procedure and home applications by the patients themselves.

Benbachir (2008) [24] applied the product used in the present study (VivaStyle Paint On Plus) on a smaller sample of patients and also found a significant difference between baseline and after treatment. Similar outcomes (whitening of 2.03 shades in canines) were obtained with the use of 5.9% hydrogen peroxide (Colgate Simply White) as bleaching agent [11], although the difference achieved by the treatment between baseline and 2 weeks was not significant when objectively measured with a spectrophotometer. Another author [25] reported a greater clinical efficacy using the same agent (5.9% hydrogen peroxide), with an improvement of 4.5 shades. The use of 6% hydrogen peroxide (Xtra White) for two weeks obtained a significant difference in mean tooth shade score with an improvement of only 1.02 shades [26]. Similar results to the present findings were also reported [27] when slightly higher concentrations of bleaching agent (8.7% hydrogen peroxide) were applied using the same paint-on gel system (Colgate Simply White Night). Studies that used 18% carbamide peroxide (Colgate Simply White Clear Whitening Gel), equivalent to 6.3% hydrogen peroxide, reported an improvement of 3.8–5.5 shades after two weeks of treatment [6, 28–30].

Differences in application procedure may explain some of the discrepancies in results. Thus, patients in our study did not close their mouths for 30 seconds after the gel application, whereas other authors instructed their patients to close their mouths immediately [11]. In some studies, the gel was applied two, three, or four times a day [21, 25, 28, 29], which may explain the greater bleaching effect achieved. 

The low efficacy found in the present study may be related to the initial shade of the teeth. The best published results [30, 31] were obtained in brown or yellow teeth (≤A3). The present study only considered teeth with values of ≥A2 because it was designed to assess the efficacy of these over-the-counter products in young adults, whose teeth frequently have light shades.

## 5. Conclusions

Application of 6% hydrogen peroxide gel with a paint-on system shows significant clinical efficacy, whether applied by a clinician in office or by the patients themselves at home.

## Figures and Tables

**Table 1:** Mean tooth shade values by group and tooth type at baseline and after 5 and 10 treatment applications.

		Baseline		5 applications		10 applications (final)	
	N	Mean	S.D.	Mean	S.D.	Mean	S.D.
GROUP I	16	7.5	1.4	6.0	1.7	4.7	1.9
GROUP II	16	7.3	1.5	5.6	1.4	4.8	1.9

## References

[1] Gerlach RW, Barker ML (2003). Randomized clinical trial comparing overnight use of two self-directed peroxide tooth whiteners. *American Journal of Dentistry*.

[2] Kishta-Derani M, Neiva G, Yaman P, Dennison J (2007). In vitro evaluation of tooth-color change using four paint-on tooth whiteners. *Operative Dentistry*.

[3] Fasanaro TS (1992). Bleaching teeth: history, chemicals, and methods used for common tooth discolorations. *Journal of Esthetic Dentistry*.

[4] Kugel G (2003). Over-the-counter tooth-whitening systems. *Compendium of Continuing Education in Dentistry*.

[5] Sagel PA, Odioso LL, McMillan DA, Gerlach RW (2000). Vital tooth whitening with a novel hydrogen peroxide strip system: design, kinetics, and clinical response. *Compendium of Continuing Education in Dentistry*.

[6] Nathoo S, Stewart B, Zhang YP, Chaknis P, Rustogi KN, DeVizio W (2002). Efficacy of a novel, nontray, paint-on 18% carbamide peroxide whitening gel. *Compendium of Continuing Education in Dentistry*.

[7] Gambarini G, Testarelli L, Dolci G (2003). Clinical evaluation of a novel liquid tooth whitening gel. *American Journal of Dentistry*.

[8] García-Godoy F, Villalta P, Bartizek RD, Barker ML, Biesbrock AR (2004). Tooth whitening effects of an experimental power whitening toothbrush relative to an 8.7% hydrogen peroxide paint-on gel control. *American Journal of Dentistry*.

[9] Slezak B, Santarpia P, Xu T, Monsul-Barnes V, Heu RT, Stranick M (2002). Safety profile of a new liquid whitening gel. *Compendium of Continuing Education in Dentistry*.

[10] Wetter NU, Branco EP, Deana AM, Pelino JEP (2009). Color differences of canines and incisors in a comparative long-term clinical trial of three bleaching systems. *Lasers in Medical Science*.

[11] Zantner C, Derdilopoulou F, Martus P, Kielbassa AM (2006). Randomized clinical trial on the efficacy of 2 over-the-counter whitening systems. *Quintessence International*.

[12] Joiner A (2004). Tooth colour: a review of the literature. *Journal of Dentistry*.

[13] Guan YH, Lath DL, Lilley TH, Willmot DR, Marlow I, Brook AH (2005). The measurement of tooth whiteness by image analysis and spectrophotometry: a comparison. *Journal of Oral Rehabilitation*.

[14] Meireles SS, Demarco FF, dos Santos IDS, Dumith SDC, Bona AD (2008). Validation and reliability of visual assessment with a shade guide for tooth-color classification. *Operative Dentistry*.

[15] da Mata AD, Da Silva Marques DN (2006). A novel technique for in-office bleaching with a 6% hydrogen peroxide paint-on varnish. *The European Journal of Esthetic Dentistry*.

[16] Ziebolz D, Hannig C, Attin T (2008). Influence of a desensitizing agent on efficacy of a paint-on bleaching agent. *American Journal of Dentistry*.

[17] Kugel G, Papathanasiou A, Williams AJ, Anderson C, Ferreira S (2006). Clinical evaluation of chemical and light-activated tooth whitening systems. *Compendium of Continuing Education in Dentistry*.

[18] Marson FC, Sensi LG, Vieira LCC, Araújo É (2008). Clinical evaluation of in-office dental bleaching treatments with and without the use of light-activation sources. *Operative Dentistry*.

[19] Hassel AJ, Cevirgen E, Balke Z, Rammelsberg P (2009). Intraexaminer reliability of measurement of tooth color by spectrophotometry. *Quintessence International*.

[20] Joiner A (2006). The bleaching of teeth: a review of the literature. *Journal of Dentistry*.

[21] Barlow A, Gerlach RW, Date RF, Brennan K, Struzycka I, Kwiatkowska A (2003). Clinical response of two brush-applied peroxide whitening systems. *Journal of Clinical Dentistry*.

[22] Gerlach RW, Barker ML (2003). Clinical response of three direct-to-consumer whitening products: strips, paint-on gel, and dentifrice. *Compendium of Continuing Education in Dentistry*.

[23] Farrell S, Barker ML, Sagel PA, Gerlach RW (2006). Use of a physical barrier to improve efficacy of a paint-on whitening gel: a seven-day randomized clinical trial. *Journal of Clinical Dentistry*.

[24] Benbachir N, Ardu S, Krejci I (2008). Spectrophotometric evaluation of the efficacy of a new in-office bleaching technique. *Quintessence International*.

[25] Gambarini G, Testarelli L, De Luca M, Dolci G (2004). Efficacy and safety assessment of a new liquid tooth whitening gel containing 5.9% hydrogen peroxide. *American Journal of Dentistry*.

[26] Collins LZ, Maggio B, Liebman J (2004). Clinical evaluation of a novel whitening gel, containing 6% hydrogen peroxide and a standard fluoride toothpaste. *Journal of Dentistry*.

[27] Sielski C, Conforti N, Stewart B (2003). A clinical investigation of the efficacy of a tooth-whitening gel. *Compendium of Continuing Education in Dentistry*.

[28] Li Y, Lee SS, Cartwright S (2004). Comparative tooth whitening efficacy of 18% carbamide peroxide liquid whitening gel using three different regimens. *Journal of Clinical Dentistry*.

[29] Brunton PA, Ellwood R, Davies R (2004). A six-month study of two self-applied tooth whitening products containing carbamide peroxide. *Operative Dentistry*.

[30] Nathoo S, Giniger M, Proskin HM (2002). Comparative 3-week clinical tooth-shade evaluation of a novel liquid whitening gel containing 18% carbamide peroxide and a commercially available whitening dentifrice. *Compendium of Continuing Education in Dentistry*.

[31] Haywood VB, Leonard RH, Nelson CF, Brunson WD (1994). Effectiveness, side effects and long-term status of nightguard vital bleaching. *The Journal of the American Dental Association*.

